# Exploring the differential mechanisms of carotenoid biosynthesis in the yellow peel and red flesh of papaya

**DOI:** 10.1186/s12864-018-5388-0

**Published:** 2019-01-16

**Authors:** Yan Hong Shen, Fei Ying Yang, Bing Guo Lu, Wan Wan Zhao, Tao Jiang, Li Feng, Xiao Jing Chen, Ray Ming

**Affiliations:** 1 College of Horticulture Science and Technology, Hebei Normal University of Science and Technology, Qinhuangdao, 066004 Hebei China; 2 College of Horticulture, Fujian Agriculture and Forestry University, Fuzhou, 350002 Fujian China; 3 College of Life Science, Fujian Normal University, Fuzhou, 350117 Fujian China; 4 FAFU and UIUC-SIB Joint Center for Genomics and Biotechnology, Haixia Institute of Science and Technology, Fujian Agriculture and Forestry University, Fuzhou, 350002 Fujian China; 5 Department of Plant Biology, University of Illinois at Urbana-Champaign, Urbana, IL 61801 USA

**Keywords:** *Carica papaya* L., Carotenoids, Metabolic pathway, RNA-Seq, Coloration

## Abstract

**Background:**

Red-fleshed papaya is a good material to study the different carotenoids accumulation mechanism in the peel and flesh. Although the peel and flesh of papaya closely integrated into one body, the flesh coloration changing from white to red, while the exocarp coloration changing from green to yellow. In this study, the major carotenoids accumulation and the expression patterns of key carotenoid biosynthesis pathway genes in the process of papaya fruit ripening were studied, and the carotenoid biosynthetic pathways in the yellow peel and red flesh of papaya were investigated.

**Results:**

The carotenoid composition in papaya flesh and peel were different. The major carotenoids were lutein and *β*-carotene in the peel, while lycopene in the flesh. The accumulation of carotenoids, including lycopene, *β*-carotene, and *β*-cryptoxanthin were considered to cause the orange-red color of papaya cv. ‘Daqing No.10’ flesh. The color of peel changed from green to yellow because of the fast degradation of chlorophyll and the appearance of carotenoids such as lutein and *β*-carotene. Thirteen genes that encode enzymes in the carotenoid biosynthetic pathway were detected in papaya fruit transcriptome: two phytoene synthase (*PSY1*, *PSY2*), two phytoene desaturase (*PDS1*, *PDS2*), one *ζ*-carotene desaturase (*ZDS*), four lycopene cyclase (*CYCB*, *LCYB1*, *LCYB2*, *LCYE*), one *β*-carotene hydroxylase (*CHYB*), one carotene *ε*-monooxygenase (*LUT1*), one violaxanthin de-epoxidase (*VDE*), and one zeaxanthin epoxidase (*ZEP*). The results of RNA-Seq and RT-qPCR showed the expression of carotenoid biosynthetic pathway genes was consistent with the change of carotenoid content. Carotenoid biosynthetic pathways in the yellow peel and red flesh of papaya were analysed based on the major carotenoids accumulation and the expression patterns of key carotenoid biosynthesis pathway genes. There was only a *β*-branch of carotenoid biosynthesis in the flesh of papaya, while there were both *α*- and *β*-branch of carotenoid biosynthesis in papaya peel. In the process of papaya fruit ripening, the *α*-branch was inhibited and the *β*-branch was enhanced in the peel.

**Conclusions:**

The differential carotenoid accumulation and biosynthesis pathway genes expression in peel and flesh, lay a foundation for further study and provide further insights to control fruit color and improve fruit quality and appearance.

**Electronic supplementary material:**

The online version of this article (10.1186/s12864-018-5388-0) contains supplementary material, which is available to authorized users.

## Background

Carotenoids are one of the most essential components for human nutrition and health, mainly due to their pro-vitamin A and antioxidant proportion. Humans are not able to synthesize carotenoids and depend entirely on natural sources or dietary supplements. Dietary carotenoids are thought to provide health benefits in decreasing the risk of disease, particularly certain cancers and eye disease [1, 2]. The most common dietary carotenoids are *α*-carotene, *β*-carotene, *β*-cryptoxanthin, lycopene, lutein, and zeaxanthin; *α*-carotene, *β*-carotene, and *β*-cryptoxanthin are precursors of vitamin A, while lycopene, lutein, zeaxanthin have no vitamin A activity but can also act as antioxidants. Besides the nutritional value of carotenoids, they are the main coloring pigments of the yellow, orange, and red colors of fruits: mango (*Mangifera indica* L.) [3], tomato (*Lycopersicon esculentum* Mill.) [4], orange (*Citrus*) [5], banana (*Musa* spp.) [6], papaya (*Carica papaya* L.) [7], and loquat (*Eriobotrya japonica* L.) [8].

The biosynthetic pathway of carotenoids in high plants has been gradually clarified by biochemical, classical genetic and molecular genetic analysis. Plant carotenoids are isoprenoid-derived molecules generally synthesized and located in plastids. Two Geranylgeranyl pyrophosphate (GGPP) molecules condense via phytoene synthase (PSY), forming phytoene; phytoene forms lycopene via phytoene desaturase (PDS) and *ζ*-carotene desaturase (ZDS); then the biosynthetic pathway of carotenoids splits after biosynthesis of lycopene into the *α*-branch and the *β*-branch: ① in *α*-branch, lycopene cyclase (LCY) catalyzes the cyclization of lycopene to form *α*-carotene; *α*-carotene is converted into lutein by *β*-carotene hydroxylase (CHYB) and carotene *ε*-monooxygenase (LUT1); ② in *β*-branch, LCY catalyzes the cyclization of lycopene to form *β*-carotene, and CHYB catalyzes the conversion of *β*-carotene to *β*-cryptoxanthin and *β*-cryptoxanthin to zeaxanthin [9, 10]. Many carotenoid synthesis genes including *PSY*, *PDS*, *ZDS*, and *LCY* have been cloned successfully and genetically modified plants with increased carotenoids levels have been obtained such as rice, tomato, potato, and canola [11, 12].

Papaya is an important tropical and sub-tropical fruit crop which is known for its high nutritional values like vitamins A and vitamins C [13, 14]. There are two types of papaya, red-fleshed and yellow-fleshed. The major carotenoid in the pulp of red-fleshed papaya is lycopene, while the major carotenoids in the yellow-fleshed papaya are *β*-carotene and *β*-cryptoxanthin [15, 16]. It has been documented that a 2-bp insertion is present in the chromoplast specific lycopene *β*-cyclase (CYCB) gene resulting in a recessive loss-of-function mutation, so as to accumulate a large amount of lycopene which is responsible for the red flesh [17]. Pigments in the flesh of papaya are known, but pigments in the peel of papaya remain unknown. The color of exocarp turns from green to yellow and the color of the flesh changes from white to red during the fruit ripening process in red-fleshed papaya. The differential accumulation of carotenoids and differential expression of carotenoid synthesis genes were studied in order to understand the different carotenoids accumulation mechanism in the peel and flesh of papaya.

## Results

### Papaya coloration during papaya fruit ripening

**Fig. 1:**
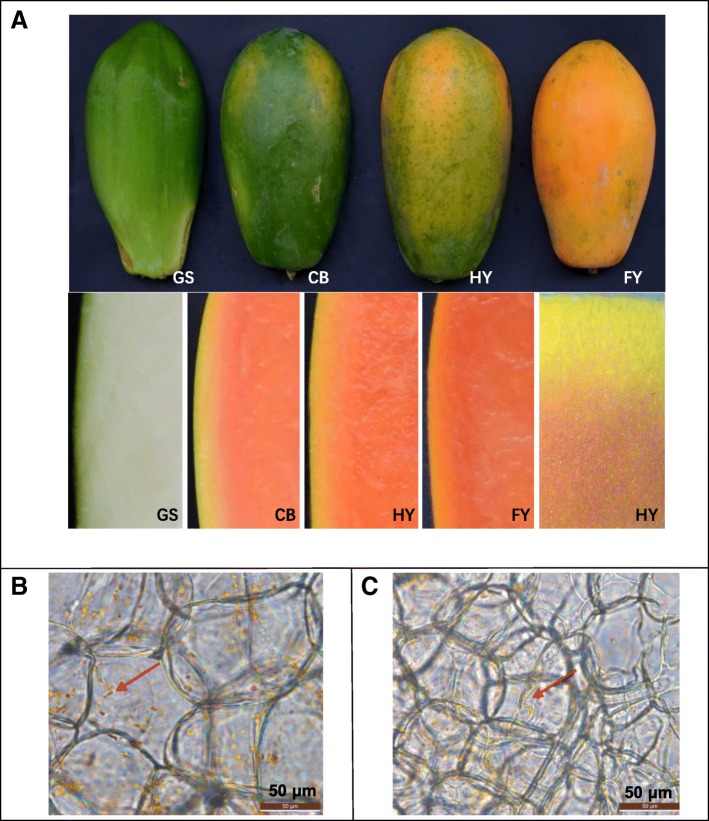
Papaya coloration during papaya fruit ripening. **a**, The color of peel and flesh at the following stages of papaya fruit development: GS, Green stage (the peel is dark green and the flesh is white); CB, Color break stage (most of the peel are still green, but there are two yellow stripes appear on the top of fruit, as well as the flesh are red); HY, Half yellow stage (the color of peel is half green and half yellow); FY, Full yellow stage (the color of peel is full yellow). **b**, The chromoplasts and flesh cells in FY stage; the red arrow in the legend indicates the red elongated contours crystals. **c**, The chromoplasts and peel cells in FY stage; the red arrow in the legend indicates the yellow round-shaped elements. Small pieces of flesh and peel of FY stage papaya were cut with a sharp razor blade to very thin sections, and the thin sections were used to observe chromoplast with an Olympus IX51 (Tokyo, Japan)

*C. papaya* cv. ‘Da Qing No.10’ is a red-fleshed papaya. During fruit ripening process, the most visible change of papaya is the color of exocarp turning from green to yellow and the color of flesh changing from white to orange-red (Fig. 1). As seen in Fig. 1a, at green stage, the peel is dark green and the flesh is white; at color break stage, most of the peel is still green, but there are two yellow stripes appear on the top of fruit, as well as the flesh is red; at half yellow stage, the color of peel is half green and half yellow; at full yellow stage, the color of peel is full yellow; the flesh and peel closely integrated into one body under the anatomical lens. The peel is not as circumscribed from the flesh. Under a microscope, the flesh cells are bigger than exocarp cells in FY red-fleshed papaya. Yellow round-shaped elements are abundant and well-distributed in peel cells with larger yellow round-shaped and several red elongated contours crystals present in flesh cells (Fig. 1b, c). Thus, the color and shape of chromoplasts have a clear difference between flesh and peel. These findings illustrated the differences in carotenoid composition between peel and flesh.

### Chlorophyll change and carotenoid accumulation

**Fig. 2:**
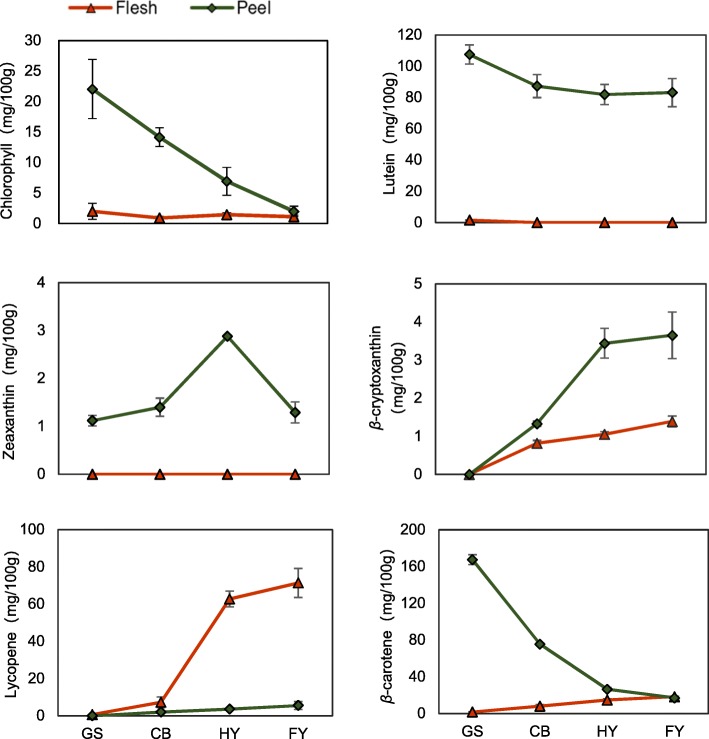
Content of major carotenoids and total chlorophyll in different papaya ripening stages. The content of total chlorophyll and major carotenoids were measured at four stages of papaya fruit development: GS, Green stage; CB, Color break stage; HY, Half yellow stage; FY, Full yellow stage. The green lines with rhombuses represent the content of chlorophyll or carotenoids in the peel, while the red lines with triangles stand for the content of chlorophyll or carotenoids in flesh. Error bars on each column indicate SDs from three replicates

The content of chlorophyll and major carotenoids was determined during the ripening of papaya fruit. The content of chlorophyll in the peel decreased during papaya ripening, and there was almost no chlorophyll detected in the flesh. The color of green stage in papaya was green because of the high contents of chlorophyll, after that the content of chlorophyll decreased sharply during the process of papaya ripening (Fig. 2). A large amount of lutein, *β*-carotene, as well as small amount of *β*-cryptoxanthin, zeaxanthin, lycopene were detected in the peel. The content of *β*-cryptoxanthin, zeaxanthin, and lycopene increased, while and the content of lutein and *β*-carotene decreased in the peel during papaya ripening. The presence of lutein, *β*-carotene, *β*-cryptoxanthin, zeaxanthin and the simultaneous rapid loss of chlorophyll caused a color shift from green to yellow during fruit ripening.

Almost no chlorophyll, lutein, and zeaxanthin were detected in the flesh, but a large amount of lycopene, some *β*-carotene, and a small amount of *β*-cryptoxanthin were detected in the flesh. In the process of fruit ripening, there was a sustained increase in carotenoids (lycopene, *β*-carotene, *β*-cryptoxanthin) content. Therefore, the flesh color changed from white to red. Except for lycopene, the other carotenoids (lutein, *β*-carotene, *β*-cryptoxanthin, zeaxanthin) content in peels were higher than that in fleshes. This illustrates that exocarp is an important source of carotenoids.

### RNA-Seq analysis

**Fig. 3:**
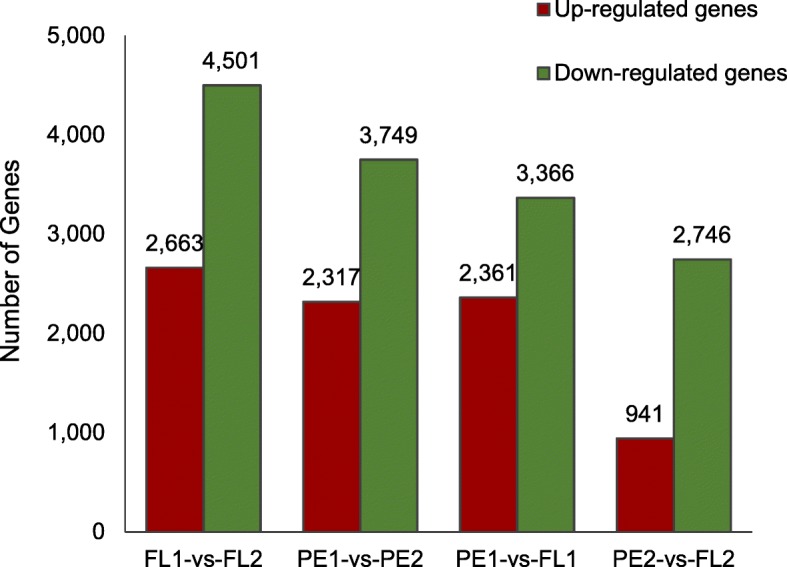
Differential expression gene statistics. The number of up-regulated and down-regulated genes between samples is summarized. The red rectangular columns represent the number of up-regulated genes while the green rectangular columns represent the number of down-regulated genes

The results of RNA-Seq are shown in Table 1 and Fig. 3. Table 1 showed the clean reads number and the gene number of the four samples. After removing low-quality reads, adaptor sequences, and rRNA reads, we obtained 44,431,496 (PE1), and 41,487,992 (PE2), 44,812,308 (FL1), 52,110,632 (FL2) clean reads. Clean reads were then mapped to the papaya reference genome. There were 18,941 (PE1), 18,242 (PE2), 18,315 (FL1), and 17,907 (FL2) expressed genes detected, with the genes in the FL2 being the least. More expressed genes at the green stage were than those at color break stage. Figure 3 showed the Differentially Expressed Genes (DEGs) in different samples. Compared with FL1, 2663 genes were up-regulated and 4501 genes were down-regulated in FL2; in papaya peel, there were 2317 genes up-regulated and 3749 genes down-regulated. This demonstrates that a large number of genes may participate in the process of fruit ripening. Comparing with the peel, the number of down-regulated genes is more than the up-regulated genes’ number in the flesh.

**Table 1:** The gene number of all samples

Sample Name	Total Clean Reads No.	Known Gene No. (Known Gene Ratio)	New Gene No.	All Gene No.	All Reference Gene No.
PE1	44,431,496	17,748 (63.91%)	1193	18,941	27,769
PE2	41,487,992	17,062 (61.44%)	1180	18,242	
FL1	44,812,308	17,141 (61.73%)	1174	18,315	
FL2	52,110,632	16,737 (60.27%)	1170	17,907	

The Known Gene Ratio = Known Gene No. / All Reference Gene No.

The pathway enrichment analysis was carried out using the KEGG pathway database. We focused on the analysis of carotenoid biosynthetic pathway. Comparing with FL1, the expression of *PDS*, *ZDS*, *CYCB* (chromoplast specific lycopene *β*-cyclase), *CHYB*, and *VDE* (Violaxanthin de-epoxidase) increased, while the expression of *LCYB2* decreased in FL2 (Additional file 1: Figure S1). Also, the expression of most carotenoid biosynthetic pathway genes (*PDS*, *ZDS*, *CYCB*, *LUT1*, *CHYB*, *VDE*, and *ZEP* (zeaxanthin epoxidase)) in the peel increased with papaya ripening, while the expression of *LCYE* decreased (Additional file 2: Figure S2). Comparing of carotenoid biosynthetic genes in the peel and flesh of papaya, we found that *LCYE* was the only gene that had been enriched in FL2 by KEGG pathway enrichment. The expression level of *LCYE* in FL2 was significantly lower than that in PE2 (Additional file 3: Figure S3). This indicated that the differential expression of *LCYE* may be the key reason for the difference of carotenoid components between flesh and peel in color break stage. As previously described, the major carotenoid in the peel was lutein, but it was not detected in the flesh. There was no lutein in the flesh because of non-detectable expression of *LCYE*.

### Screening carotenoid biosynthesis related genes in the transcriptome

**Fig. 4:**
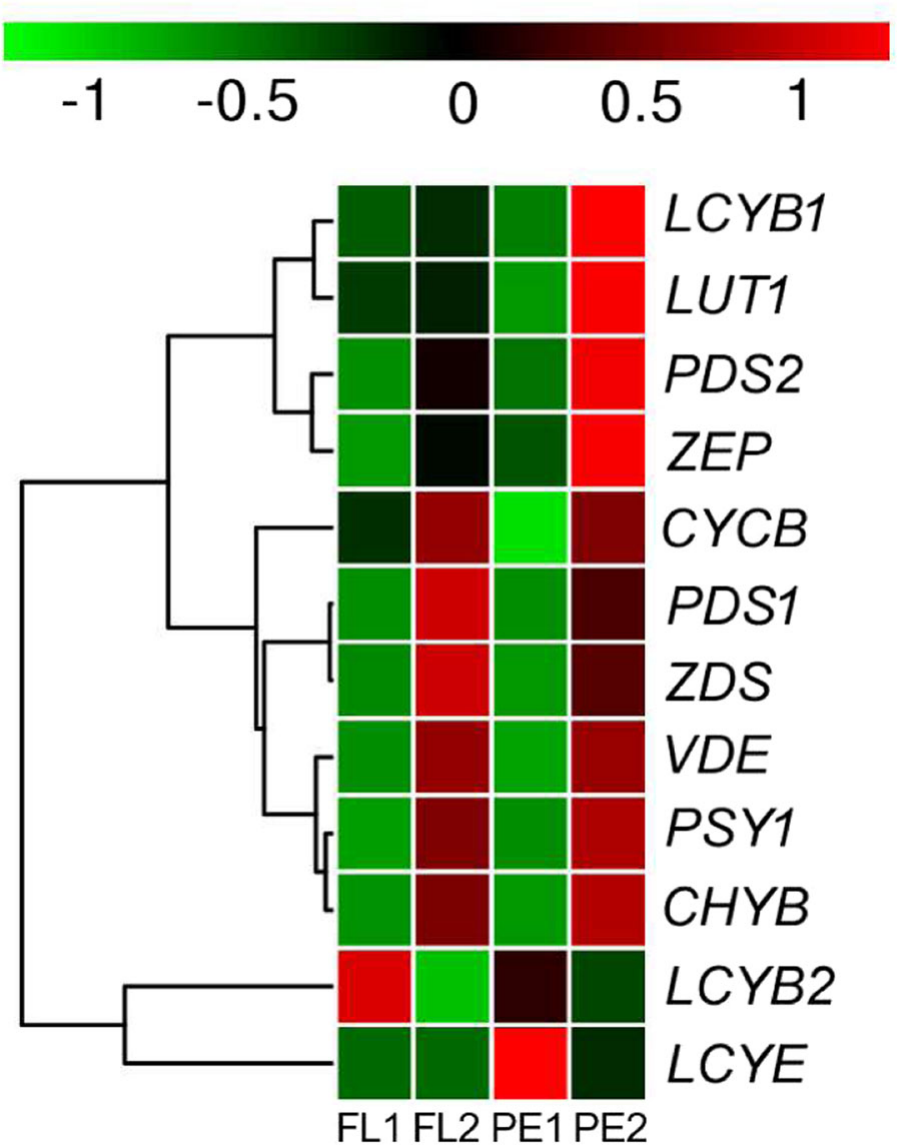
Heat map diagram of expression levels for carotenoid biosynthetic pathway genes analyzed by KEGG. The heat map was drawn according to FPKM values. Columns and rows in the heat map represent samples and genes, respectively. Sample names are displayed below the heat map. Color scale indicates fold changes in gene expression

We analyzed the genes involved in carotenoid biosynthesis. Thirteen papaya genes that encode enzymes in the carotenoid biosynthetic pathway were detected in papaya transcriptome: two phytoene synthase (*PSY1*, *PSY2*), two phytoene desaturase (*PDS1*, *PDS2*), one *ζ*-carotene desaturase (*ZDS*), four lycopene cyclase (*CYCB*, *LCYB1*, *LCYB2*, *LCYE*), one *β*-carotene hydroxylase (*CHYB*), one carotene *ε*-monooxygenase (*LUT1*), one violaxanthin de-epoxidase (*VDE*), and one zeaxanthin epoxidase (*ZEP*) (Additional file 4: Table S1). The heat map of the carotenoid biosynthesis related genes was drawn according to FPKM (Fragments Per Kilobase of transcript per Million mapped reads) values (Fig. 4). *LCYB2* and *LCYE* were closely related in a cluster, while the other ten genes are in a big cluster (Fig. 4). Except for *LCYB2* and *LCYE*, the expression of most carotenoid biosynthetic pathway genes (*PDS*, *ZDS*, *CYCB*, *LUT1*, *CHYB*, *VDE*, and *ZEP*) increased during papaya ripening.

Phytoene synthase (*PSY*) is a key regulator in the carotenoid biosynthetic pathway [18]; Two *PSY* genes were detected in the transcriptome. The expression of *PSY1* increased in the flesh and peel, and no expression of *PSY2* was detected, thereby demonstrating that *PSY1* was active in the carotenoid biosynthetic pathway in papaya. Similar to papaya, *PSY1* plays an important role in tomato fruit tissue pigmentation too [19]. Two *PDS* genes were detected, and the expression patterns were the same, but the expression abundance between the two genes was different. The expression of *PDS1* in FL2 is 394.46, while that of *PDS2* is 7.9 (Additional file 4: Table S1). Because the abundant gene transcripts account for more enzymatic activity participating in carotenoid synthesis, *PSY1* and *PDS1* are the major genes involved in the synthesis of carotenoid in fruit. The expression of two genes (*ZDS* and *CHYB*) increased with papaya ripening. Four lycopene cyclase genes were detected: *LCYB1*, *LCYB2*, *CYCB,* and *LCYE*. *LCYB1* and *CYCB* had been cloned and characterized previously [20, 21]. We didn’t analyze *CYCB* because there is a 2-bp insertion in *CYCB* gene in red-fleshed papaya, *CYCB* is a functional gene in yellow-fleshed papaya, but a pseudo gene in red-fleshed papaya [17]. The expression of the other three lycopene cyclase genes was different. This showed that they may play different roles in carotenoid synthesis.

### RT-qPCR analysis of transcription levels of five carotenoid biosynthetic pathway genes

**Fig. 5:**
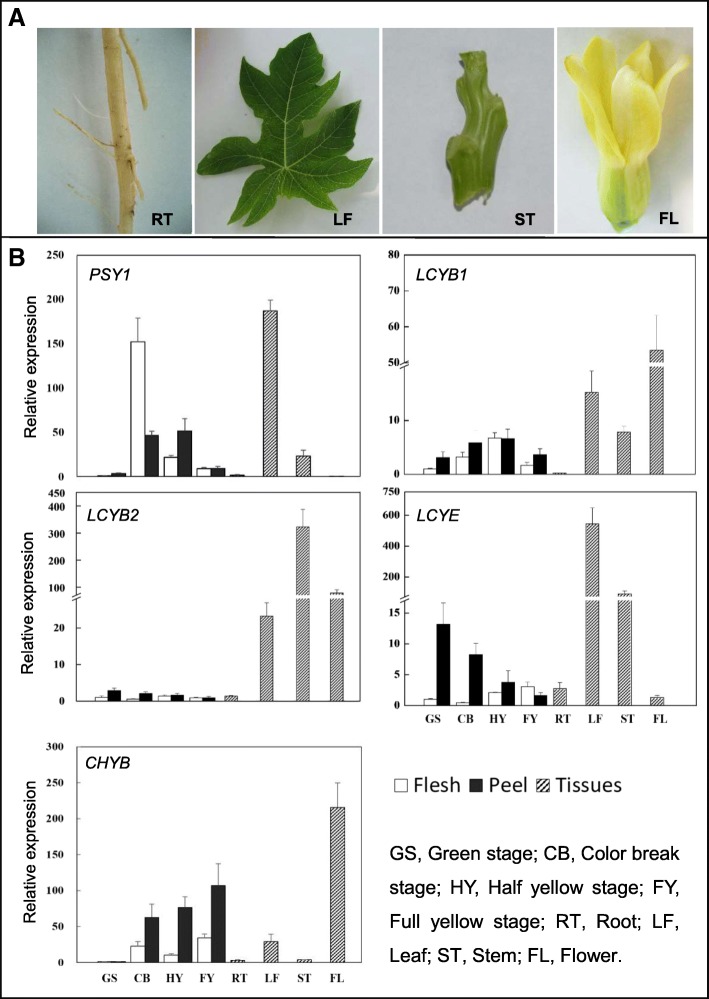
Transcription levels of five carotenoid biosynthetic pathway genes in different organs. **a**, The pictures of different organs were used in this study. **b**, The relative transcript levels of *PSY1*, *LCYB1*, *LCYB2*, *LCYE*, *CHYB* in root, leaf, stem, flower, and four developmental stages of papaya fruit were measured through RT-qPCR. RT-qPCR was performed with gene-specific primer sets in Table 2. Expression data were normalized to the expression of *CpActin*. Error bars on each column indicate SDs from three replicates

The expression of five key carotenoid biosynthetic pathway genes *PSY1*, *LCYB1*, *LCYB2*, *LCYE*, *CHYB*, in different ripening papaya fruits and different organs was studied using RT-qPCR analysis. The transcript levels of *PSY1* increased first and then decreased with papaya ripening (Fig. 5b). The expression pattern of *PSY1* was similar in the flesh and peel. *LCYB1* also expressed in both flesh and peel, and the transcript levels increased first then decreased too. The expression of *LCYB2* and *LCYE* in the peel were higher than that in the flesh, and the transcript levels were decreased with papaya ripening. The transcript level of *CHYB* was higher in the peel than that in the flesh, and the *CHYB*’s expression presented a trend of rising. Figure 5b also shows the results of the relative transcript levels of the five carotenoid biosynthesis-related genes in different organs (root, leaf, stem, and flower). *PSY1* expressed in leaf, stem, and ripening fruit; *LCYB1* expressed in leaf, stem, fruit, flower, while almost no expression was detected in the root; *LCYB2* has a higher expression in leaf, stem, and flower, a lower expression in fruit. *LCYE* transcripts predominate in green color tissues, such as green peel, leaf, and stem.

### Carotenoid biosynthetic pathway in the yellow peel and red flesh of papaya

**Fig. 6:**
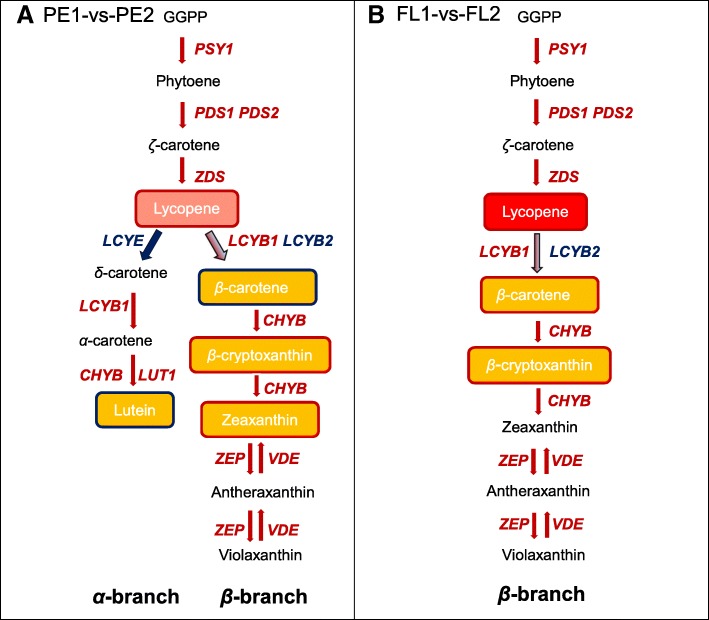
Carotenoid biosynthetic pathway in the yellow peel and red flesh of papaya. **a**, Carotenoid biosynthetic pathway in the sample of PE2 versus PE1 (PE1-vs-PE2). **b**, Carotenoid biosynthetic pathway in the sample of FL2 versus FL1 (FL1-vs-FL2). Genes examined are in red or blue italic bold letters: genes with red or blue color means they are up- or down-regulated during papaya fruit ripening respectively. The carotenoids in rectangular boxes were determined in the study. The red-frame boxes refer to the content of carotenoid is increasing during papaya fruit ripening and the blue-frame boxes refer to the content of carotenoid is decreasing. (The diagram is modified from Naik et al. [40])

Based on the major carotenoids accumulation and the expression patterns of key carotenoid biosynthesis pathway genes, the carotenoid biosynthetic pathway in the yellow peel and red flesh of papaya were speculated (Fig. 6). There were both *α*- and *β*-branch of carotenoid biosynthesis in the peel of papaya, while there was only *β*-branch in the flesh. A large amount of lutein, *β*-carotene, as well as small amount of *β*-cryptoxanthin, zeaxanthin, lycopene were in the peel. The most abundant carotenoid in the flesh was lycopene, followd by *β*-carotene and *β*-cryptoxanthin. In the peel, the key carotenoid biosynthesis pathway genes: *PSY1*, *PDS1*, *ZDS*, *LCYB1*, *CHYB*, *LUT1*, *ZEP*, and *VDE* were up-regulated during papaya fruit ripening, while *LCYE* and *LCYB2* were down-regulated; in the flesh, *PSY1*, *PDS1*, *ZDS*, *LCYB1*, *CHYB*, *ZEP*, and *VDE* were also up-regulated during papaya fruit ripening.

## Discussion

Although the flesh and peel of red-fleshed papaya fruit are closely integrated into one body, the coloration of the flesh and peel are quite different: the color of exocarp turning from green to yellow and the color of flesh changing from white to red. So exploring the differential mechanisms of carotenoid biosynthesis in the yellow peel and red flesh of papaya is an interesting research point. The color transformation is directly related to the change of the carotenoid component. Some studies showed that the concentration of carotenoids was significantly higher in the peel than that in the flesh such as *Citrus iyo* fruit [22] and loquat fruit [8]. The coloration of loquat peel due to the transient increase of carotenoids and degradation of chlorophylls [8]. In papaya fruit, carotenoids (lycopene, *β*-carotene, *β*-cryptoxanthin) accumulates extensively in the flesh, therefore the flesh color changes from white to red; although the content of lutein and *β*-carotene decreased in the peel, the rapid loss of chlorophyll leads to the appearance of carotenoids causes a color shift from green to yellow. Microscopic observation also proved that the carotenoid composition between the peel and the flesh are different. Yellow round-shaped elements are abundant in peel cells, while larger yellow round-shaped and several red elongated contours crystals present in flesh cells. This result is consistent with previous research that *β*-carotene is deposited in globular and tubular elements in a lipid-dissolved form whereas lycopene is deposited in a solid crystalline form [23, 24].

The synthesis of carotenoid in peel and flesh of papaya had been analysed base on the distribution of carotenoid components and the expression of carotenoid biosynthesis pathway genes. The increase in the content of *β*-cryptoxanthin and zeaxanthin was accompanied by the decrease in the content of lutein and *β*-carotene, indicating that there were both *α*- and *β*-branch in the peel and the *β*-branch was enhanced in the process of papaya fruit ripening. However, there is only a *β*-branch in the flesh because no lutein and expression of *LCYE* were detected, *LCYE* was the only gene that had been enriched in FL2 by KEGG pathway enrichment. It expressed abundantly in the green peel, but hardly expressed in the flesh. *LCYE* is the key reason for the differential carotenoid accumulation in the flesh and peel of papaya. Except for *LCYE*, three lycopene cyclases had been detected in papaya fruit transcriptome, *LCYB1*, *CYCB*, and *LCYB2*. *LCYB1* has been considered as a chloroplast-specific lycopene *β*-cyclase as well as *CYCB* is a chromoplast-specific lycopene *β*-cyclase [20, 21]. Although the gene of *CYCB* was named as *LCY-β2* in previous studies, *CYCB* was used in our paper [20, 25]. *LCYB1* was supposed to be a chloroplast-specific lycopene *β*-cyclase, but it had higher expression level in red flesh and the downstream products of lycopene *β*-cyclase like *β*-carotene and *β*-cryptoxanthin were also detected, which means *LCYB1* expresses in non-chlorophyllous tissue as well. This is not consistent with previous studies [20]. *LCYB2* is a newly discovered gene of lycopene cyclase in papaya, the expression of *LCYB2* in peel was higher than that in flesh, and the transcript levels had been decreasing with papaya fruit ripening. However, the function of *LCYB2* is still unclear in papaya.

The reason for the conversion from *α*-branch to *β*-branch in the peel of papaya during fruit ripening is not clear. One of the key roles of carotenoids in plants is protecting the photosynthetic apparatus against photoinhibition and photodamage [26]. The xanthophyll cycle transforms the excitation energy into heat and thereby prevents the formation of damaging ROS. It is an important photoprotective process in plants [27]. Violaxanthin de-epoxidase (VDE) and zeaxanthin epoxidase (ZEP) are the two enzymes in the xanthophyll cycle. In excess light conditions, VDE catalyzes the conversion of violaxanthin to zeaxanthin via antheraxanthin, whereas ZEP catalyzes the reverse reaction [14]. The expression of *ZEP* and *VDE* were up-regulated in color break stage of papaya, this may due to chlorophyll degradation enhanced the activity of the xanthophyll cycle to avoid severe photodamage under strong illumination.

By above knowable, the differential expression of the key carotenoid biosynthesis pathway genes in the peel and flesh of papaya result in the differential accumulation of carotenoids. But little is known about the regulatory mechanisms of the genes involved in *ɑ*- and *β*-branch. Transcriptional factors (TFs) play important roles in a variety of cellular and developmental processes, such as fruit ripening, senescence, hormone signalling, and various biotic and abiotic stress responses. For example, several TFs have been reported to be involved in the ripening of banana [28], tomato [29], and papaya [30]. Recently, some TFs were found to play essential roles in regulating the carotenoid biosynthesis in plants. An *R2R3-MYB* represses the transformation of *α*- and *β*-branch carotenoids by negatively regulating expression of *CrBCH2* and *CrNCED5* in flavedo of *Citrus* reticulate [31]. *CpNAC1* is a positive regulator of carotenoid biosynthesis during papaya fruit ripening [32]. The protein of CpNAC2 physically interacts with CpEIN3a, which act as a transcriptional activator of *CpPDS4* and *CpCHY-b* by directly binding to their promoters has been proposed [33]. Several TFs including *MYB*, *ERF*, *bZIP*, *bHLH*, *WRKY*, and *NAC* had been selected according to the expression patterns with papaya fruit ripening in our research (data not given). Some TFs expression decreased in both flesh and peel, while some TFs were gradually increased, paralleling the increase of the carotenoids content; some TFs have higher expression levels in the flesh, and some TFs have much higher expression levels in the peel. The differential expression of these TFs suggests that they may be involved in the regulation of carotenoid biosynthesis genes transcription in flesh or peel. The functions of these TFs should be studied in the future.

## Conclusions

Carotenoid biosynthetic pathway in the yellow peel and red flesh of papaya was revealed on the basis of the major carotenoids accumulation and the expression patterns of key carotenoid biosynthesis pathway genes. There is only a *β*-branch of carotenoid biosynthesis in the flesh of papaya, while there are both *α*- and *β*-branch in papaya peel. However, additional genomics wide analysis are required to determine and validate. This work elucidated the differential anabolic pathway of carotenoids in peel and flesh of papaya, and provide further insights to control fruit color and improve fruit quality and appearance.

## Methods

### Plant materials

Papaya fruits (*C. papaya* cv. ‘Daqing No.10’) in different development stages (GS, Green stage; CB, Color break stage; HY, Half yellow stage; FY, Full yellow stage) were harvested from Longzhijia fruit and vegetable farmers’ specialized cooperatives in November in Zhangzhou, China. The healthy fruits of similar size, shape, and maturity were divided into four groups. Five papaya in the same stage were peeled, seeds were removed, and the peel and flesh were cut into some pieces respectively. The pieces were mixed and weighed, frozen in liquid nitrogen, and stored at − 80 °C. Finally, eight types of samples were obtained, namely FL1 (the flesh of GS), FL2 (the flesh of CB), FL3 (the flesh of HY), FL4 (the flesh of FY), PE1 (the peel of GS), PE2 (the peel of CB), PE3 (the peel of HY), PE4 (the peel of FY). Tissue samples (root, stem, leaf, and flower) were collected for reverse transcription quantitative PCR (RT-qPCR) analysis. For chromoplast observation, small pieces of flesh and peel of FY stage papaya were obtained respectively. All pieces were cut with a sharp razor blade to thin sections. Then the ultrathin sections were put on a clean slide and examined with an Olympus IX51 (Tokyo, Japan).

### Spectrophotometric determination of chlorophyll content

Chlorophyll was extracted from 200 mg of ground papaya fruit materials using 25 mL of 80% acetone. The absorbance was determined at 663 nm and 646 nm with a UV-Vis spectrophotometer equipped with 1.0 cm quartz cells. Based on the equation of total chlorophyll concentration CT = 7.18 × A663 + 17.32 × A646, total chlorophyll content was determined [34].

### UPLC analysis of major carotenoids

An modified method for the extraction of major carotenoids from papaya fruit was used in the study [35]. Briefly, the sample (peel or flesh) was lyophilized using a freeze dryer at − 40 °C for two days, followed by mechanical grinding with liquid nitrogen, and stored at − 20 °*C. papaya* fruit (250 mg) was added into 2.5 mL of acetone (1:10). Ultrasonic-assisted extraction was used to extract carotenoids for 1 h at 240 W at 20 °C, and then centrifuged for 10 min at 10,000 rpm at 4 °C. The liquid supernatant was filtered by 0.22 μm organic filter, and stored at − 20 °C for later use.

Carotenoid pigments analysis was referenced to Yan [35] and Lee [36]. Chromatography was carried out with a Waters Ultra-high performance liquid chromatography. Samples were measured by a C_18_ carotenoid column (100 mm × 2.1 i.d., 1.7 μm) from Waters. The eluent was methyl tertiary butyl ether and methanol (*V/V* = 30:70) with the type of isocratic elution. And each eluent contained 0.01% BHT and 0.05% TEA (triethylamine) as modifiers in order to prevent the degradation of carotenoids on the column. The following rate was 0.5 mL/min, column temperature was 25 °C, and the injection volume was 5 μL, analyzing with UV at 445 nm. The lutein, *β*-carotene, lycopene standards were obtained from Solarbio (Beijing Solarbio Science & Technology Co., Ltd., Beijing, China). The *β*-cryptoxanthin standard was from Sigma (USA).

### RNA extraction and RNA-Seq

Total RNA was extracted from the papaya fleshes or peels using an RNA extraction kit (Dongsheng Biotech, Guangzhou, China). Total RNA of four samples (PE1, PE2, FL1, FL2) was used to prepare cDNA libraries using the Illumina Dynabeads® mRNA DIRECT™ Kit. Then, the cDNA libraries were used for paired-end 125 sequencing using an Illumina Hiseq2500 at Genedenovo Biotechnology Co., Ltd. (Guangzhou, China). In total, four sets of raw reads were obtained, and all sequencing data were deposited in the NCBI Sequence Read Archive (SRA). The raw reads were filtered to remove “dirty” data, including low-quality reads, adaptor sequences, and rRNA reads, to generate “clean” reads. FPKM (Fragments Per Kilobase of transcript per Million mapped reads) was used to measure the transcript abundance of each gene. Those with a fold-change of ≥2 and a false discovery rate (FDR) < 0.05 were considered significantly differentially expressed genes (DEGs) [37]. All of the genes were annotated using the reference papaya genome database (*Carica papaya* Version1.181: CpGDB181(JGI)) [38], NCBI non-redundant (Nr) database, the Kyoto Encyclopedia of genes and genomes (KEGG) pathway database. The KEGG enrichment analysis was performed with a Qvalue cut off of 0.05.

### Reverse transcription quantitative PCR analysis

For RT-qPCR, oligonucleotide primers were designed according to each gene’s 3′-untranslated region with DNAMAN (Table 2). *CpActin* was used as the reference gene. RT-qPCR was carried out using SYBR Green-based PCR assay in a Bio-Rad CFX96 Real-Time PCR System (Bio-Rad, USA). Each reaction mix contained 1.0 μL of cDNAs, SYBR Premix ExTaq™ 10 μL, PCR forward primer (10 μmol·L^− 1^) 0.5 μL, PCR reverse primer (10 μmol·L^− 1^) 0.5 μL, ddH_2_O 8.0 μL, to a final volume of 20 μL. The PCR conditions were 95 °C for 3 min, followed by 40 cycles of 95 °C for 15 s, 56 °C for 30 s, and 72 °C for 20 s. Each RT-qPCR analysis was performed in triplicate, and the mean was used for RT-qPCR analysis. The relative expression of the genes was calculated according to the method of 2^−△△Ct^ [39], and SPSS was used to analyze the data.

**Table 2:** PCR primers used in this study

Primer	Sequence(5′–3′)
*PSY1-F*	TGATGACTGAGGCTTATGACC
*PSY1-R*	CCACCTATCTAAAGCTGTTGG
*LCYB1-F*	ACTTGCTAGACTGCCTTGAC
*LCYB1-R*	ACAGTGGCCTGAATCGTAAC
*LCYB2-F*	CTATGCAAGAGACCCAACAAG
*LCYB2-R*	GAGCTCCTTCCTTGAGATGTTC
*LCYE-F*	GGCTCCAAAGTATGCTTCAGT
*LCYE-R*	CCAGTCAGGTAGGTGGAAGAAAG
*CHYB-F*	CTCTCCGCCGCCATTACC
*CHYB-R*	TCCTCCAACACGAAGCAGAC
*Actin-F*	CACCGAGGCTCCACTTAACC
*Actin-R*	ACCAGAGTCCAGCACAATACC

## Additional files

Additional file 1: **Figure S1**. KEGG graph of carotenoid biosynthetic pathway (FL1-vs-FL2). 1.3.5.5 indicates PDS (evm.TU.supercontig_157.3, fold 4.4); 1.3.5.6 indicates ZDS (evm.TU.supercontig_117.67, fold 4.3); CrtL-b indicates CYCB (evm.TU.supercontig_195.16, fold 1.0); CruA/P indicates LCYB2 (evm.TU.supercontig_132.5, fold − 1.6); CrtR indicates CHYB (evm.TU.supercontig_107.106, fold 3.2); 1.23.5.1 indicates VDE (evm.TU.supercontig_51.78, fold 1.4). (DOCX 51 kb)

Additional file 2: **Figure S2**. KEGG graph of carotenoid biosynthetic pathway (PE1-vs-PE2). 1.3.5.5 indicates PDS (evm.TU.supercontig_157.3, fold 3.9); 1.3.5.6 indicates ZDS (evm.TU.supercontig_117.67, fold 5.3); CrtL-e indicates LCYE (evm.TU.supercontig_28.134, fold − 2.5); CrtL-b indicates CYCB (evm.TU.supercontig_195.16, fold 5.4); LUT1(evm.TU.supercontig_5.131, fold 1.1); CrtR indicates CHYB (evm.TU.supercontig_107.106, fold 3.9); 1.23.5.1 indicates VDE (evm.TU.supercontig_51.78, fold 1.6); 1.14.1390 indicates ZEP (evm.TU.supercontig_55.146, fold 1.4). (DOCX 50 kb)

Additional file 3: **Figure S3**. KEGG graph of carotenoid biosynthetic pathway (PE2-vs-FL2). CrtL-e indicates LCYE (evm.TU.supercontig_28.134, fold − 3.9). (DOCX 50 kb)

Additional file 4: **Table S1**. Carotenoid biosynthesis-related genes. (DOCX 16 kb)

## Abbreviations

CB: Color break stage
CHYB: *β*-carotene hydroxylase
CYCB: Chromoplast-specific lycopene *β*-cyclase
DEGs: Differentially expressed Genes
DGE: Differential gene expression
FL1: The flesh of GS
FL2: The flesh of CB
FL3: The flesh of HY
FL4: The flesh of FY
FPKM: Fragments Per Kilobase of transcript per Million mapped reads
FY: Full yellow stage
GS: Green stage
HY: Half yellow stage
KEGG: Kyoto Encyclopedia of Genes and Genomes
LCYB: Lycopene *β*-cyclase
LCYE: Lycopene *ε*-cyclase
LUT1: Carotene *ε*-monooxygenase
PDS: Phytoene desaturase
PE1: The peel of GS
PE2: The peel of CB
PE3: The peel of HY
PE4: The peel of FY
PSY: Phytoene synthase
RT-qPCR: Reverse transcription quantitative PCR
SRA: Sequence Read Archive
TEA: Triethylamine
TFs: Transcription factors
UPLC: Ultra-high Performance Liquid Chromatography
VDE: Violaxanthin de-epoxidase
ZDS: *ζ*-carotene desaturase
ZEP: Zeaxanthin epoxidase
