# Supplementary material for: Exploring the differential mechanisms of carotenoid biosynthesis in the yellow peel and red flesh of papaya
Source: BMC Genomics. 2019 Jan 16;20:49. doi: 10.1186/s12864-018-5388-0 (PMC6335806; doi:10.1186/s12864-018-5388-0)
Supplement: Supplementary file 1 — Figure S1. KEGG graph of carotenoid biosynthetic pathway (FL1-vs-FL2). 1.3.5.5 indicates PDS (evm.TU.supercontig_157.3, fold 4.4); 1.3.5.6 indicates ZDS (evm.TU.supercontig_117.67, fold 4.3); CrtL-b indicates CYCB (evm.TU.supercontig_195.16, fold 1.0); CruA/P indicates LCYB2 (evm.TU.supercontig_132.5, fold − 1.6); CrtR indicates CHYB (evm.TU.supercontig_107.106, fold 3.2); 1.23.5.1 indicates VDE (evm.TU.supercontig_51.78, fold 1.4). (DOCX 51 kb) [file 12864_2018_5388_MOESM1_ESM.docx]

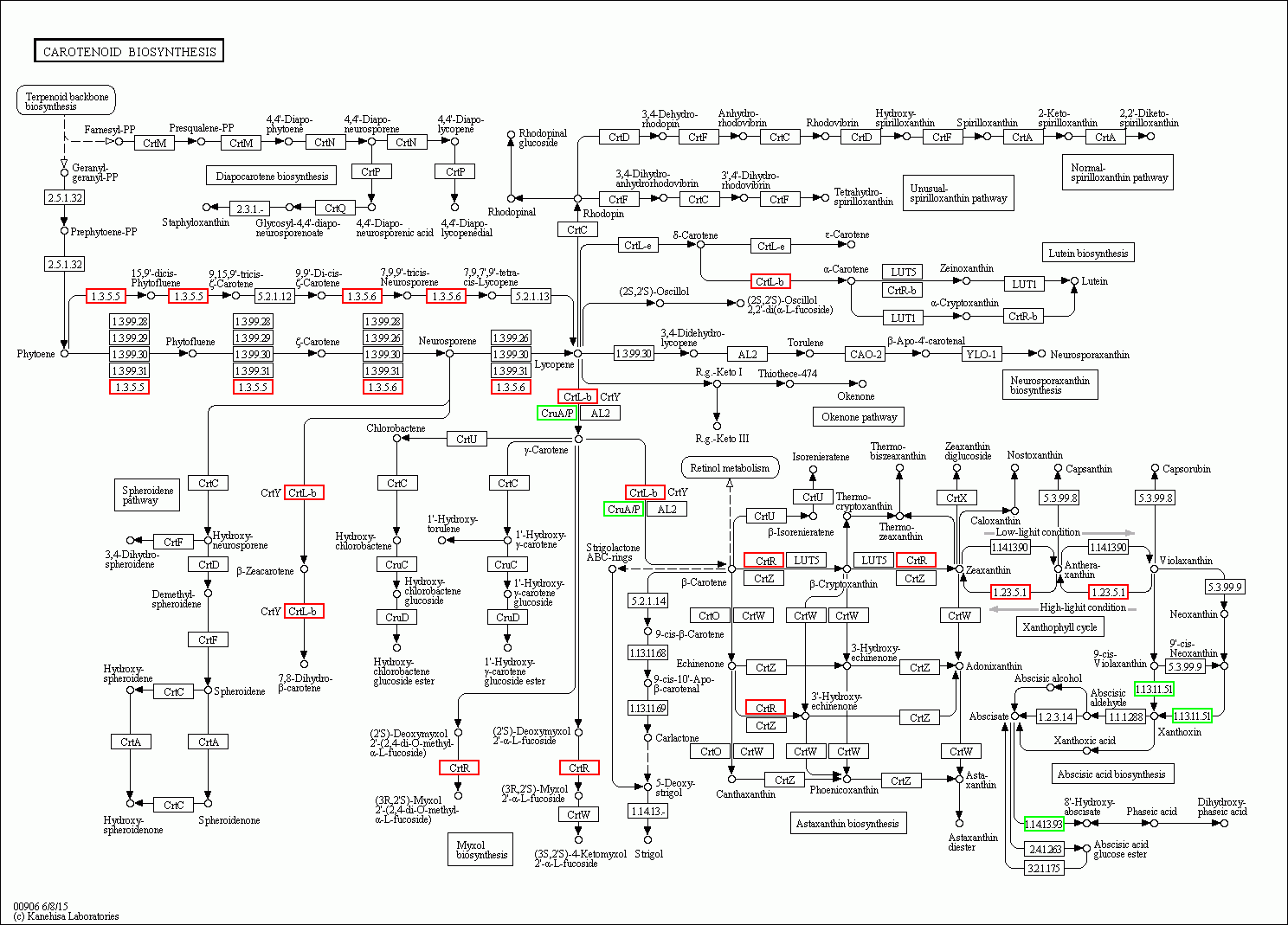


**Additional figure 1** KEGG graph of carotenoid biosynthetic pathway (FL1-vs-FL2).

1.3.5.5 indicates PDS (evm.TU.supercontig_157.3, fold 4.4);

1.3.5.6 indicates ZDS (evm.TU.supercontig_117.67, fold 4.3);

CrtL-b indicates CYCB (evm.TU.supercontig_195.16, fold 1.0);

CruA/P indicates LCYB2 (evm.TU.supercontig_132.5, fold -1.6);

CrtR indicates CHYB (evm.TU.supercontig_107.106, fold 3.2);

1.23.5.1 indicates VDE (evm.TU.supercontig_51.78, fold 1.4 ).
