# Supplementary material for: Exploring the differential mechanisms of carotenoid biosynthesis in the yellow peel and red flesh of papaya
Source: BMC Genomics. 2019 Jan 16;20:49. doi: 10.1186/s12864-018-5388-0 (PMC6335806; doi:10.1186/s12864-018-5388-0)
Supplement: Supplementary file 2 — Figure S2. KEGG graph of carotenoid biosynthetic pathway (PE1-vs-PE2). 1.3.5.5 indicates PDS (evm.TU.supercontig_157.3, fold 3.9); 1.3.5.6 indicates ZDS (evm.TU.supercontig_117.67, fold 5.3); CrtL-e indicates LCYE (evm.TU.supercontig_28.134, fold − 2.5); CrtL-b indicates CYCB (evm.TU.supercontig_195.16, fold 5.4); LUT1(evm.TU.supercontig_5.131, fold 1.1); CrtR indicates CHYB (evm.TU.supercontig_107.106, fold 3.9); 1.23.5.1 indicates VDE (evm.TU.supercontig_51.78, fold 1.6); 1.14.1390 indicates ZEP (evm.TU.supercontig_55.146, fold 1.4). (DOCX 50 kb) [file 12864_2018_5388_MOESM2_ESM.docx]

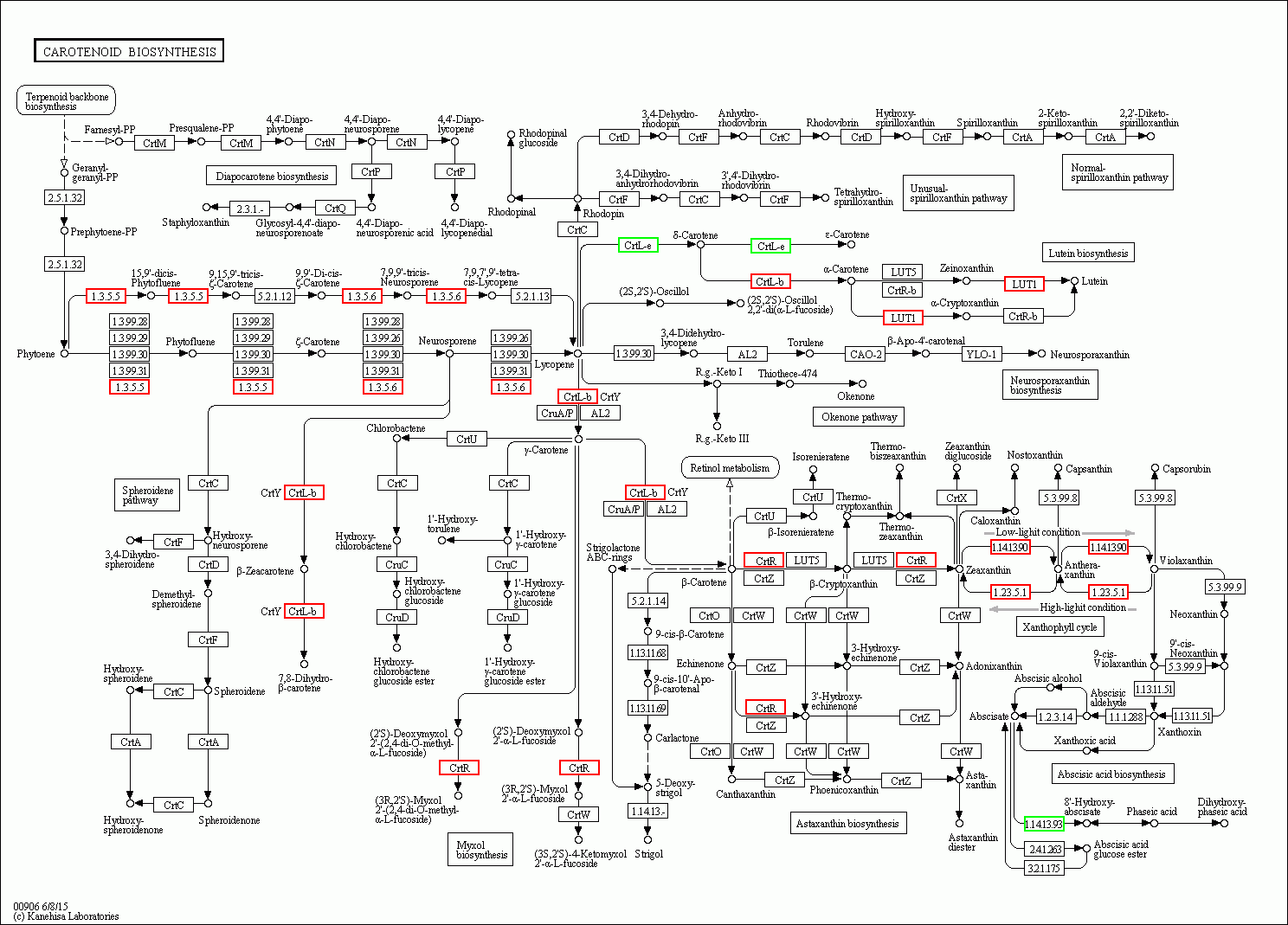


**Additional figure 2** KEGG graph of carotenoid biosynthetic pathway (PE1-vs-PE2).

1.3.5.5 indicates PDS (evm.TU.supercontig_157.3, fold 3.9);

1.3.5.6 indicates ZDS (evm.TU.supercontig_117.67, fold 5.3);

CrtL-e indicates LCYE (evm.TU.supercontig_28.134, fold -2.5);

CrtL-b indicates CYCB (evm.TU.supercontig_195.16, fold 5.4);

LUT1 (evm.TU.supercontig_5.131, fold 1.1);

CrtR indicates CHYB (evm.TU.supercontig_107.106, fold 3.9)；

1.23.5.1 indicates VDE (evm.TU.supercontig_51.78, 1.6);

1.14.1390 indicates ZEP (evm.TU.supercontig_55.146, fold 1.4）
