# Supplementary material for: Exploring the differential mechanisms of carotenoid biosynthesis in the yellow peel and red flesh of papaya
Source: BMC Genomics. 2019 Jan 16;20:49. doi: 10.1186/s12864-018-5388-0 (PMC6335806; doi:10.1186/s12864-018-5388-0)
Supplement: Supplementary file 3 — Figure S3. KEGG graph of carotenoid biosynthetic pathway (PE2-vs-FL2). CrtL-e indicates LCYE (evm.TU.supercontig_28.134, fold − 3.9). (DOCX 50 kb) [file 12864_2018_5388_MOESM3_ESM.docx]

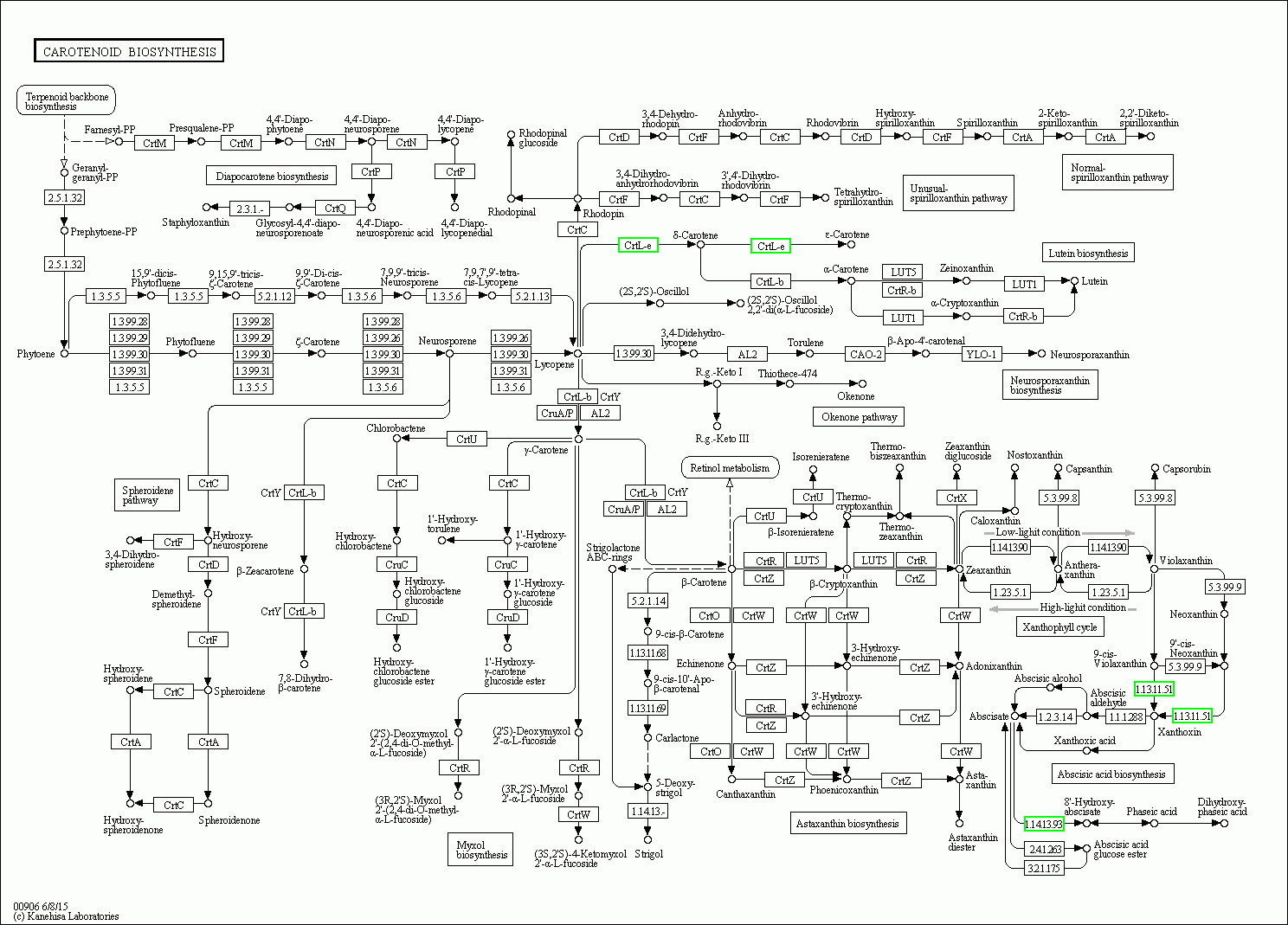


**Additional figure 3** KEGG graph of carotenoid biosynthetic pathway (PE2-vs-FL2).

CrtL-e indicates LCYE ( evm.TU.supercontig_28.134, fold -3.9)
