# Supplementary material for: Exploring the differential mechanisms of carotenoid biosynthesis in the yellow peel and red flesh of papaya
Source: BMC Genomics. 2019 Jan 16;20:49. doi: 10.1186/s12864-018-5388-0 (PMC6335806; doi:10.1186/s12864-018-5388-0)
Supplement: Supplementary file 4 — Table S1. Carotenoid biosynthesis-related genes. (DOCX 16 kb) [file 12864_2018_5388_MOESM4_ESM.docx]

**Additional table 1** Carotenoid biosynthesis-related genes

| **No** | **Gene ID**  **(evm.TU.supercontig_)** | **Definition** | **Symbol** | **FL1_**  **fpkm** | **FL2_**  **fpkm** | **PE1_**  **fpkm** | **PE2_**  **fpkm** |
| --- | --- | --- | --- | --- | --- | --- | --- |
| 1 | 43305.1 | Phytoene synthase | PSY1 | 5.78 | 137.27 | 13.78 | 158.02 |
| 2 | 119.76 | Phytoene synthase | PSY2 | 0.00 | 0.00 | 0.00 | 0.00 |
| 3 | 157.3 | Phytoene desaturase 3 | PDS1 | 18.71 | 394.46 | 16.81 | 252.24 |
| 4 | 20.108 | Phytoene desaturase 3 | PDS2 | 3.44 | 7.90 | 4.25 | 14.18 |
| 5 | 117.67 | *ζ*-carotene desaturase | ZDS | 51.34 | 998.10 | 16.74 | 662.26 |
| 6 | 195.16 | Chromoplast-specific lycopene *β*-cyclase | CYCB | 5.30 | 10.83 | 0.24 | 10.36 |
| 7 | 5.198 | Lycopene *β*-cyclase | LCYB1 | 13.41 | 16.21 | 11.37 | 33.9 |
| 8 | 132.5 | Lycopene *β*/*ε*-cyclase protein isoform 2 | LCYB2 | 9.52 | 3.09 | 6.78 | 5.02 |
| 9 | 28.134 | Lycopene *ε*-cyclase | LCYE | 0.20 | 0.11 | 9.03 | 1.62 |
| 10 | 107.106 | *β*-carotene hydroxylase | CHYB | 9.60 | 90.94 | 7.34 | 108.02 |
| 11 | 5.131 | Carotene *ε*-monooxygenase | LUT1 | 11.30 | 11.94 | 9.08 | 18.87 |
| 12 | 51.78 | Violaxanthin de-epoxidase | VDE | 17.72 | 46.70 | 15.88 | 47.30 |
| 13 | 55.146 | Zeaxanthin epoxidase | ZEP | 9.18 | 18.00 | 13.57 | 35.10 |
